# The effect of interprofessional education on interprofessional professionalism behaviors of the surgical team members

**DOI:** 10.1186/s12912-022-01015-9

**Published:** 2022-08-25

**Authors:** Azam Hosseinpour, Fatemeh Keshmiri, Sara Jambarsang, Fatemeh Jabinian, Seyed Mostafa Shiryazdi

**Affiliations:** 1 Department of Operating Room, School of Allied Medical Sciences, Qom University of Medical Sciences, Qom, Iran; 2 Medical Education Department, Education Development Center, Shahid Sadoughi University of Medical Sciences, Yazd, Iran; 3 Center for Healthcare Data Modeling, Departments of Biostatistics and Epidemiology, Shahid Sadoughi University of Medical Sciences, Yazd, Iran; 4 Department of Operating Room and Anesthesiology, School of Allied Medical Sciences, Shahid Sadoughi University of Medical Sciences, Yazd, Iran; 5 General Surgery Department, Medical School, Shahid Sadoughi University of Medical Sciences, Yazd, Iran

**Keywords:** Interprofessional, Professionalism, Medical, Collaboration, Excellence, Respect, Interprofessional Professionalism, Interprofessional Professionalism assessment, Surgery

## Abstract

**Introduction:**

Interprofessional professionalism (IPP) has been introduced as one of the critical sub-competencies of interprofessional collaboration. This study aimed to assess the effect of interprofessional education on the behavior of interprofessional professionalism among the surgical team in the intervention compared to the control group.

**Methods:**

This is a quasi-experimental study. The participants were nurses in anesthetist and surgical technology and surgical residents of Shahid Sadoughi Hospital (*n* = 150) who were included in the study by the census. The intervention employed an interprofessional case-based learning strategy to explore themes of interprofessional professionalism. Two assessors used the Interprofessional Professionalism Assessment (IPA) tool to measure learners’ performance while observing them in practice prior to the intervention, one and three months after the intervention. Data were analyzed using descriptive tests (mean and SD) and RM-ANOVA.

**Results:**

In this study, the participants in the intervention (*n* = 78) and the control (*n* = 72) groups entered the study. The Baseline IPA scores of participants were reported as 1.25 (0.12) and 1.21 (0.1) in the intervention and control groups, respectively. The IPA score of the participants in the intervention group (2.59 (0.26) and 2.54 (0.24)) was higher than the control group (1.17 (0.08) and 1.12 (0.07)) after one and three months of the intervention (*P* = 0.0001). The effect of educational interventions was reported at the large level (Eta Square = 0.89).

**Conclusion:**

Interprofessional professionalism in surgical teams has been recognized as a critical element of team-based care. The present study used an interprofessional education strategy to develop IPP behavior. All professions benefited from interprofessional education. It is suggested that all surgical team professionals participate in interprofessional education.

**Supplementary Information:**

The online version contains supplementary material available at 10.1186/s12912-022-01015-9.

## Introduction

Interprofessional Professionalism (IPP) focuses on the professional aspects of teamwork [1] and explains the values and principles of professionalism in interprofessional collaboration [2]. IPP is categorized as a subset of professionalism and emphasizes working with people from different professions to demonstrate mutual respect and shared values. The IPP includes respect for the humanism and dignity of other professions, attention to the values of teamwork, responsibility, accountability, and management of interprofessional challenges [2].

Surgical units have high-stress environments where team members must work together to provide safe care [3]. Surgical teams of healthcare professionals with different expertise require contributing with other team members as partners in achieving a common goal. Improving IPP among the health team members is recognized as an influential element in achieving interprofessional cooperation in the operating room [4]. It is essential to define specific standards of professional behavior based on the surgery fields [4]. The University of Southern California has defined a framework of professionalism in surgery. The framework comprises 11 attributes: clinical competence, cultural competence, altruism, leadership, accountability, interpersonal skills, respect, practice improvement, ethics/legal, appearance, and education [5].

Creating and maintaining teamwork and interprofessional collaboration in a multidisciplinary surgery team (consisting of surgeons, anesthesiologists, nurses, anesthetists, and surgery technology nurses) with different personalities is a complex task [3, 6]. Challenges with multi-discipline teams may negatively affect team performance and patient care [3, 7]. Lack of attention to professionalism in interprofessional cooperation can cause tension in relationships, reduce job satisfaction, and increase team members’ stress [8].

The World Health Organization has suggested interprofessional education as a critical strategy for preparing healthcare workers to respond to healthcare needs by collaboration [9]. “*Interprofessional education* is defined as when learners in different professions’ learn about, from, and with each other [9].” Interprofessional education prepares learners to work together in future careers [9]. A BEME guide highlights studies showing the effectiveness of interprofessional education (IPE) on attitude and knowledge, but limited studies have examined the effects of IPE on the performance of IPP [10]. The results of a systematic review pointed out that there is no consensus on effective professionalism education in surgery, and further studies are needed in this field [11]. The present study was conducted to compare the effects of an interprofessional educational intervention on the control vs. intervention groups on observed IPP and determine any changes over time in the intervention group.

## Methods

**fig. 1:**
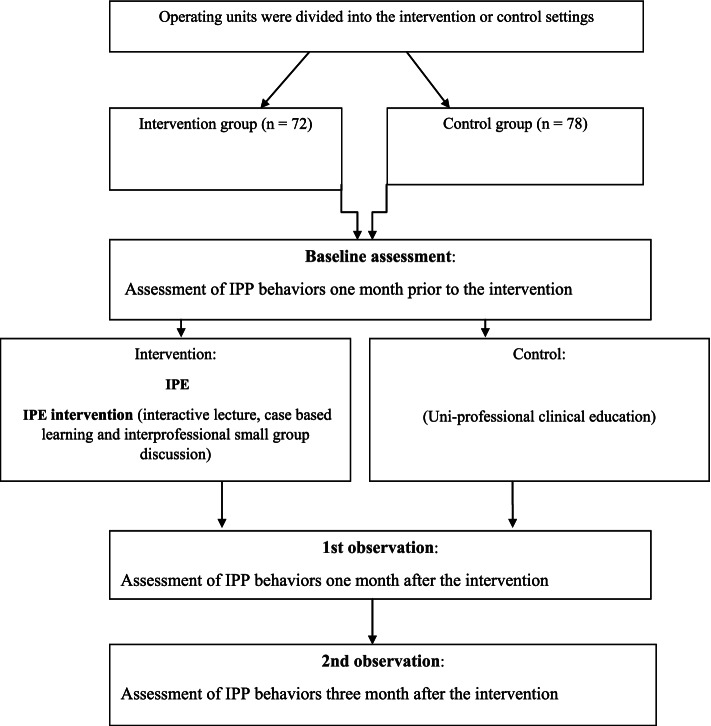
Flow chart of the study steps

The present study was a quasi-experimental design. This design has been recognized as a proper design in educational research at times when random assignment and matching of participants to an intervention and comparison groups is not possible [12, 13]. Figure 1 illustrates the current study design.

### Setting

The present study was conducted at Shahid Sadoughi University of Medical Sciences in 2020–2021. In our context, surgical physicians, surgical residents, and nurses in surgical technology and anesthetics participated in surgical teams. Clinical education was just used as a uni-professional strategy in the context. In addition, the planned professionalism education is considered in no clinical education programs for different professions.

### Participants

In this study, 150 participants were divided into intervention (*n* = 78) and control (*n* = 72) groups. Different professions represented were surgical technology nursing, anesthetics nursing, and residences of surgical specialties in general surgery, ENT, ophthalmology, and orthopedics that work in the operating units of Shahid Sadoughi hospital. Four operating units in the hospital, and their respective surgical staff, were used for this study and divided into the intervention and control groups. Those assignments were made random. Accordingly, two units were assigned to the intervention group and the other two to the control.

The demographic information of participants in the intervention and control groups showed in Table 1.

**Table 1:** Demographic characteristic of participants

	Intervention group *N* (%)	Control group *N* (%)
**Professions**		
Surgical residents	32(21.33)	30(20)
Surgical technology nurses	28(18.66)	26(17.33)
Anesthesia nurses	18(12)	16(10.66)
**Gender**		
Male	33(22)	30(20)
Female	45(30)	42(28)
**Age**	35.68 (7.62)	34.35 (5.36)

### Educational intervention

The components of the educational intervention are explained in Table 2.

### Interactive lecture

In the first training session, an interactive lecture helped participants become familiar with the concepts of interprofessional professionalism, values, and cooperation.

### Case-based learning (CBL)

In the preparation phase of CLB, the cases were developed based on the principles of creating good cases [14]. Criteria such as reality, alignment with educational objectives, cases’ educational values, and content coverage were considered. Interprofessional critical incidents in communication, respect, altruism, and excellence were deliberated. The educational cases (*N* = 18) were developed by clinical teachers (*n* = 3) in surgery, surgical technology nursing, anesthetics nursing, and expert in health professions education (*n* = 1). The validity of cases was reviewed by experts in a panel of clinical teachers from different professions who had working experience in surgical teams (*n* = 8). The CBL is conducted in seven steps: presentation of a case, analysis and discussion by the interprofessional group, formulation of learning objectives related to IPP, dividing topics among students, reviewing the literature and educational materials by self-study, sharing of opinions among the participants [15].

In the implementation phase of CBL, participants were divided into ten interprofessional small groups. The educational cases were presented at first. The participants were asked to identify ethical challenges, recognize possible causes, and find suggested solutions to solve ethical challenges in interprofessional teams. They experienced the cased-based learning in the seven steps. Finally, the participants discussed and summarized their findings among participants in all groups. Two faculty members facilitated the steps.

### Interprofessional small group discussion

The step aimed to share the experienced issues related to IPP among different professions. The participants were asked to reflect on the experienced IPP challenges and share them with other group members. The members discussed the possible causes of the mentioned challenges and proposed their solutions to solve them.

Each interprofessional group in the mentioned methods included 3–4 surgical residents and 4–5 nurses. Two faculty members in medical education and medical ethics who were experts in professionalism facilitated the group activities and directed their discussion.

Over six months, participants met six times: 1^st^ meetings = interactive lecture and interprofessional small group discussion, 2^nd^-6^th^ meetings = interprofessional case-based learning.

### Learner/intervention assessment

Two clinical teachers who were affiliated with the surgical departments participated as assessors. The assessors with no working experience in the studies units were selected to control bias based on assessors’ working relationships with some subjects. Moreover, in order to control bias in assessment, we used different methods that recommended to control of subjectivity [16, 17] such as using two evaluators, training evaluators, evaluating in multiple situations in the surgery units, using a specific checklist, determining the way of scoring and agreeing on how the scoring of checklist’s items. Our study held two educational sessions for the assessor’s training. The session was facilitated by an expert in *Health Professions Education* who had experience in professionalism, interprofessional professionalism, and interprofessional collaboration. In the first sessions, the assessors discussed the IPP concepts and domains. The items of interprofessional professionalism assessment (IPA), the scoring method of the instrument, and the principles of observational evaluation such as time and frequency were argued in the sessions. In the second session, the assessors discuss the practical description of IPA items and their scoring to enhance their agreement about items. Afterward, we showed a video about interprofessional teamwork in the surgical unit. The video displayed a healthcare team’s performance in managing a patient with Hip fraction. The assessors were asked to evaluate the performance of members of the team (a surgical physician and a surgical nurse). This was an exercise in a simulated situation. Afterward, the facilitator debated assessment results with the assessors, and individual feedback was provided to them. In order to ensure the reproducibility of the evaluations, the evaluators’ agreement coefficients were calculated. (Kappa = 0.87).

In order to assess the IPP behaviors of the participants in the intervention and control groups, each assessor completed the IPA form after observing the participants’ performance (at least after three observations) in the operating rooms. The assessors observed each participant one month prior to the intervention. In addition, participants’ performances were assessed twice after the educational interventions (one month and three months later).

The original IPA was designed and validated by Frost et al., and included two sections: 1) demographic questions, and 2) 26 behavioral items representing six domains of Interprofessional Professionalism (RMSEA = 0.055, 90% CI: 0.042 – 0.067; CFI = 0.991; SRMR = 0.030)[18]. Authors recommended revision and further testing of a shortened instrument using 18 of the items representing 4 domains; altruism, excellence, respect, and communication (RMSEA = 0.064, 90% CI: 0.055– 0.078; CFI = 0.991; SRMR = 0.027 and Cronbach’s alpha = 0.96) [18]. The present study used the 18-item version of the instrument with demographic questions of age, gender, and profession. The validity and reliability of the questionnaire were approved in the investigated context. (CVR = 0.71, CVI = 0.91, Cronbach’s alpha = 0.83 and ICC = 0.74). Scoring on each item is on a scale of 1–5 for the competency scores where poor = 1, moderate = 2, good = 3, very good = 4, excellent = 5. The mean score of the items in each domain was reported. The IPA was validated in the previous study. (Cronbach’s alpha coefficient = 0.83 and ICC = 0.74). [19].

### Control group

the participants in the control group participated in separate classes conducted uni-professionally (*n* = 3 sessions). The classes are conducted by an expert in medical ethics and professionalism. The educational content was related to the code of conduction of professionalism and IPP. A lecture helped participants become familiar with the concepts of interprofessional professionalism and the domains. After that, the lecturer discussed the positive and negative cases of professionalism and IPP.

### Data analysis

The descriptive statistics, Mean (SD) of the total score, and number (%) were used to describe the data. For each participant, five scores were calculated: one means item score over each of the four domains and one grand mean score over all the items. All mean scores were between 1–5.

Based on Table 3, comparisons were made between control and intervention groups for the baseline data and both post-intervention observations. The group comparison was conducted after adjusting the demographic variables, including age, gender, and profession. To compare the crude and adjusted scores of IPP domains across the study (baseline measurements, after one month and after three months), used the RM-ANOVA test (Repeated Measure of Variance). The effect of intervention groups over times on scores of domains including altruism, excellence, respect, and communication was calculated. The results showed the minimum *p*-value of interactions was 0.22 that was not significant. Partial eta-squared (η2) was used for effect size calculations in RM-ANOVA. Based on a general ‘rule of thumb,’ the effect sizes are considered small, medium, and significant effects if the calculated partial η2 are approximately equal to 0.01, 0.06, and 0.14, respectively [20]. The significance is considered at *p* < 0.05.

**Table 2:** The components of educational intervention

Educational objective	The objective of intervention was to improve interprofessional professionalism behaviors of participants in different domains: altruism, excellence, respect, communication
Educational strategy	Interprofessional education
Teaching and learning methods	Interactive learning Case based learning Interprofessional small group discussion
Educational content	*Concepts:* The educational content was developed based on reports Interprofessional Education Collaborative, Interprofessional Professionalism Collaborative, IPA and contextualized guidelines of professional behavior (1–4) *Educational cases*: The cases addressed the challenges of inappropriate communication and accepting each other’s opinions (4 cases), individualistic and disrespectful relationships (3 cases), the challenges of excellence and interprofessional learning at work (4 cases) and weakness of altruism and empathy (3 cases)
Assessment	Assessment of interprofessional professionalism behavior of participants by the observational evaluation

## Results

The results showed that the domains of IPA and total scores in the intervention group were significantly higher than in the control group. Furthermore, after adjusting the demographic variables, these scores were significantly higher in the intervention group. (Table 3). The effect of educational interventions was Eta Square = 0.89 that categorized at a large level. (Table 3).

**Table 3:** The participants’ scores of interprofessional professionalism behaviors in the intervention and control groups before and after the intervention

**Domains**	**Group**	**Pre-Test**	**1**^**ST**^** Post- Test**	**2**^**nd**^** Post-Test**	Crude *P*-Value*	Adjusted** *P*-Value	**Eta-Square**
		Mean(SD)	Mean(SD)	Mean(SD)			
**Communication**	Intervention	1.27 (0.19)	2.68 (0.31)	2.62 (0.29)	< 0.001	< 0.001	0.85
	Control	1.16 (0.17)	1.22 (0.16)	1.14 (0.12)			
**Respect**	Intervention	1.14 (0.13)	2.65 (0.30)	2.61 (0.29)	0.004	< 0.001	0.87
	Control	1.18 (0.23)	1.19 (0.15)	1.16 (1.16)			
**Altruism And Empathy**	Intervention	1.51 (0.23)	2.46 (0.32)	2.40 (0.28)	0.027	< 0.001	0.82
	Control	1.43 (0.24)	1.19 (0.16)	1.13 (0.11)			
**Excellence**	Intervention	1.11 (0.11)	2.56 (0.29)	2.52 (0.27)	< 0.001	< 0.001	0.87
	Control	1.07 (0.11)	1.09 (0.09)	1.07 (0.09)			
**Total**	Intervention	1.25 (0.12)	2.59 (0.26)	2.54 (0.24)	< 0.001	< 0.001	0.89
	Control	1.21 (0.1)	1.17 (0.08)	1.13 (0.07)			

^*^ Baseline measurements adjusted Comparison between intervention and control groups based on Repeated Measure ANOVA test (RM-ANOVA) is significant at the level of 0.05. The *p*-value of interaction in RM-ANOVA was reported

^**^ Adjusted on age, sex, profession and baseline measurements

## Discussion

The development of professionalism has been recognized as one of the influential factors in developing interprofessional collaboration among health care teams [21]. Improving interprofessional professionalism and team performance is vital to providing safe care in stressful fields such as surgical units [22]. The results indicated that the IPP behavior scores of the participants of the interprofessional interventions were higher than the control group. The educational effect of the interprofessional interventions was reported to a large level.

Training methods such as case-based learning, problem-based method, and group discussion are recommended for teaching ethical values in the interprofessional approach [23–25]. These methods help the learner analyze the subjects, identify problems, compare and evaluate the desired solutions, develop decision-making on ethical problems and improve professional behaviors [26, 27]. In the present study, the educational intervention provided an interactive situation for participants in different professions were contributed in the small interprofessional groups. They discussed their opinion about the IPP dimensions and challenges. Consistency, the structured discussion, is recognized as a valuable tool for teaching ethics and professionalism [28]. The critical method of the present study was case-based learning in small interprofessional groups. The methods were designed based on the principles of participatory learning, which provides the situations for active participation of learners in interprofessional discussions. The educational cases illustrated authenticity situations, which provide opportunities to express opinions and conflicts with other team members and resolve them. It seems that holding interprofessional discussions has led to the recognition of the attitudes and opinions of others and has improved communication and respect among team members. Interprofessional education reduces defensive and hostile behaviors, negative stereotypes, ethical conflicts, and contradictions by promoting interaction between learners of different health professions [29]. In addition, interprofessional discussion resulted in a better understanding of interprofessional differences and the improvement of mutual trust and respect among team members [3, 30, 31].

Results showed that interprofessional intervention has a large educational effect on the IPP behavior of participants. The IPP behaviors of participants in the intervention group were reported to be higher than the control group. A systematic review by Guraya and colleagues indicated that interprofessional education promotes learners’ better understanding of the values and importance of other professions and strengthens participatory learning in the interprofessional environment. These improved effective working relationships among different health professions. More studies addressed the effect of education on learners’ cognitive abilities (reasoning, comprehension, and knowledge) while improving behavior as the main expected outcome less have been considered in studies [32]. The Manspeaker’s results indicated enhanced student confidence and understanding of interprofessional competencies resulting from using case-based learning in ethics workshops [25]. Wilhelm’s study showed that interprofessional ethics education increases students’ understanding of interprofessional ethical decisions [33]. The impact of interventions for students who start the process earlier – during professional education may have different outcomes compared to professionals who worked in the system.

The need for developing IPP behavior among all professions as a vital element for interprofessional collaboration was emphasized [7, 11]. Respect, effective communication, altruism, and excellence are crucial elements of IPP, which were investigated in the present study [18]. “Communication” as a critical competency of surgery team members [34, 35] consists of active listening, respectful communication, cooperation with other health team members, and accountability to the needs of other colleagues [18]. A review study found that group discussion as an interactive method is one of the most common teaching methods to improve communication in operating and surgical units [36]. The present results showed that the participants’ scores in the communication domain were significantly higher in the intervention group than in the control group and had the highest score among other domains. The interprofessional small group method provided situations for discussion about challenging interprofessional situations and the roles on the team members. The methods changed learners’ attention to professional principles and increased interactions and interprofessional communication among team members.

“Excellence” is a critical domain of professionalism that emphasizes the development of personal and professional capabilities [37]. The excellence domain consists of the use of evidence and the opinion of others in the process of patient management, collaboration with other team members to develop a suitable care plan, and clarification of ambiguous information [18]. The present results showed that the participants’ scores in the excellence domain in the intervention were significantly higher than in the control group. The present study showed that interprofessional education effectively creates a sense of empathy and intimacy among members of different professions. This strategy has changed behaviors related to excellence and interprofessional learning among participants. Similarly, Sprung’s study showed that knowledge of the characteristics of excellence could help students and young physicians recognize the features of an excellent physician and strive to achieve them [37].

“Respect for others” has been identified as a critical element of professionalism [38]. Respect for human dignity and profession in teamwork is critical to effective relationships in healthcare teams [3]. The respect domain examined the cultural differences between various health disciplines and respecting other health team members’ values, opinions, and expertise, explaining their roles and responsibilities and understanding them [18]. The present results showed that the participants’ scores in the ‘respect’ domain were significantly higher than in the control group. Kaldeim’s study showed that mutual respect is crucial in team members’ social relationships, making them feel valued [3]. Cunningham’s study found that interprofessional education in a clinical setting allows students to better understand the roles of health care professionals through active learning. Participants in this study stated that interprofessional discussion leads to better recognition of the roles and responsibilities of the others and respect for each other in patient care, which is consistent with the present results [31].

“Altruism,” as a critical concept of interprofessional professionalism, focuses on empathy, and understanding of the needs and values of others, preferring the patient’s needs to others and helping team members [18, 30, 38]. Developing altruism in interprofessional collaboration is difficult but necessary for team-based care [30]. The present results showed that the score of participants who contributed to the interprofessional education was significantly higher than the control group. Interprofessional education by providing an interactive and participatory situation influenced the altruism of learners in the intervention group.

### Study limitations

The present study was quasi-experimental, involving limitations such as a non-equivalent control group. Performing the study at only one institution can also impede generalizability. Another limitation could be the use of only two assessors for the evaluation of participants’ behavior.

## Conclusion

Interprofessional professionalism in surgical teams has been recognized as a critical element of team-based care. The present study used an interprofessional education strategy to develop IPP behavior. The educational effect of the intervention was reported at a large level. The results showed that the IPA scores of participants in the intervention group were significantly higher than in the control group. All professions benefited from interprofessional education. It is suggested that all surgical team professionals participate in interprofessional education.

## Supplementary Information

**Additional file 1: Appendix 1: **Interprofessional professionalism assessment (IPA).

## Data Availability

The datasets used and/or analyzed during the current study are available from the corresponding author on reasonable request.
