# Supplementary material for: The effect of interprofessional education on interprofessional professionalism behaviors of the surgical team members
Source: BMC Nurs. 2022 Aug 25;21:239. doi: 10.1186/s12912-022-01015-9 (PMC9414088; doi:10.1186/s12912-022-01015-9)
Supplement: Supplementary file 1 — Additional file 1: Appendix 1: Interprofessional professionalism assessment (IPA). [file 12912_2022_1015_MOESM1_ESM.docx]

| **Appendix 1: Interprofessional professionalism assessment (IPA)** |
| --- |
| 1. Works with members of other health professions to coordinate communication with patients/clients and family members. |
| 1. Demonstrates active listening with members of other health professions. |
| 1. Communicates respectfully with members of other health professions. |
| 1. Communicates with members of other health professions in a way they can understand, without using profession-specific jargon. |
| 1. Responds to questions posed by members of other health professions in a manner that meets the needs of the requester. |
| 1. Recognizes that other health professions may have their distinct cultures and values, and shows respect for these. |
| 1. Respects the contributions and expertise of members of other health professions. |
| 1. Seeks to understand the roles and responsibilities of members of other health professions as related to care. |
| 1. Determines patient care roles and responsibilities in a respectful manner with members of other health professions. |
| 1. Offers help to members of other health professions while caring for patients. |
| 1. Demonstrates empathy for members of other health professions. |
| 1. Models for other health professionals in terms of showing sympathetic behavior towards patients/clients, families and caregivers. |
| 1. Prefers patient/client needs to those of his/her own needs and other health professionals. |
| 1. Coordinates with other health professions and the patient/client, family and caregivers to produce an optimal plan of care. |
| 1. Reviews all relevant documentation from other health care professions prior to making recommendations to plan for care. |
| 1. Contributes to decisions on patient care regardless of hierarchy/profession-based boundaries. |
| 1. Works with members of other health professions to ensure continuity of care for patients. |
| 1. Seeks clarification from members of other health professions about unclear information. |
| This questionnaire was developed by Forst and colleagues(1). |

1. Frost JS, Hammer DP, Nunez LM, Adams JL, Chesluk B, Grus C, et al. The intersection of professionalism and interprofessional care: development and initial testing of the interprofessional professionalism assessment (IPA). Journal of interprofessional care. 2019;33(1):102-15.
